# Immunomodulation by maternal autoantibodies of the fetal serotoninergic 5-HT_4 _receptor and its consequences in early BALB/c mouse embryonic development

**DOI:** 10.1186/1471-213X-7-34

**Published:** 2007-04-19

**Authors:** Rehab Kamel, Simone Garcia, Frank Lezoualc'h, Rodolphe Fischmeister, Sylviane Muller, Johan Hoebee, Pierre Eftekhari

**Affiliations:** 1 CNRS UPR 9021 Immunologie et Chimie Thérapeutiques Institut de Biologie Moléculaire et Cellulaire, 15, rue René Descartes, Strasbourg, 67084, France; 2 Fdn Oswaldo Cruz, Salvador de Bahia, Brazil; 3 INSERM, U769, Signalisation et Physiopathologie Cardiaque, Châtenay-Malabry, 92296 France; 4 Université Paris Sud, Faculté de Pharmacie, IFR-141, Châtenay-Malabry,92296 France; 5 FTD, Institut de Pharmacologie, Faculté de Médicine, Strasbourg, F-67000, France

## Abstract

**Background:**

The presence of functional 5-HT_4 _receptors in human and its involvement in neonatal lupus erythematosus (NLE) have prompted us to study the receptor expression and role during embryogenesis. Earlier we managed to demonstrate that female BALB/c mice immunized against the second extracellular loop (SEL) of the 5-HT_4 _receptor gave birth to pups with heart block. To explain this phenomenon we investigated the expression of 5-HT_4 _receptors during mouse embryogenesis. At the same time we looked whether the consequence of 5-HT_4 _receptor immunomodulation observed earlier is in relation to receptor expression.

We studied the expression of 5-HT_4 _receptor at the mRNA level and its two isoforms 5-HT_4(a) _and 5-HT_4(d) _at the protein level in embryos from BALB/c mice, at 8^th^, 12^th^, 18^th ^gestation days (GD) and 1 day post natal (DPN). Simultaneously the receptor activity was inhibited by rising antibodies, in female mice against SEL of the receptor. The mice were mated and embryos were collected at 8^th^, 12^th^, 18^th ^GD and 1 DPN.

**Results:**

5-HT_4 _receptor mRNA increased in brain from 12^th ^GD to 1 DPN. Its expression gradually decreased in heart and disappeared at birth. This was consistent with expression of the receptor isoforms 5-HT_4(a) and (d)_. Abnormalities like decreased number of embryos, growth delay, spina bifida and sinus arrhythmia from 12^th ^GD were documented in pups of mice showing anti-5-HT_4 _receptor antibodies.

**Conclusion:**

serotoninergic 5-HT_4 _receptor plays an important role in mouse foetal development. In BALB/c mice there is a direct relation between the expression of receptor and the deleterious effect of maternal anti-5-HT_4 _receptor autoantibodies in early embryogenesis.

## Background

Serotoninergic 5-HT_4 _receptors belong to the family of 7-membrane spanning receptors coupled to G_s _protein. Until now 10 different human isoforms have been cloned and sequenced (h5-HT_4(a), (b), (c), (d), (e), (f), (g), (hb), (i) and (n)_) [1-3] They are all coded by a complex gene (700 Kb, 38 exons), which generates 9 carboxy-terminal variants issued from alternative splicing. They have identical sequences up to Leu^358^, with the exception of h5-HT_4(hb)_, which is characterized by a 14 amino acid residues insertion within the receptor second extracellular loop (SEL) of h5-HT_4(b) _[4]. The presence of 5-HT_4 _receptor has been reported in different tissues. Receptor mRNA has been detected in brain, bladder, gastrointestinal tract, heart, kidney, pancreas and testis [3,5,6]. In postmortem human brain studies the receptor, ignoring its different isoforms, was detected in the basal ganglion (caudate nucleus, putamen, nucleus accumbens, globus pallidus and substantia nigra), in cortex and hippocampus (CA1 and subiculum) [7]. In heart, mRNA for different h5-HT_4 _isoforms is mainly detected in atrium (5-HT_4(a), (b), (c), (g), (i)_) [3,5]. The presence of four isoforms, mainly m5-HT_4(a), (b), (e), (f) _has been described in mouse [8,9]. Although many studies have been performed to highlight the tissue distribution and functional activity of different isoforms of the receptor in adult human, rat and mouse, little is known about their expression and function during embryogenesis. Previously we have reported the involvement of 5-HT_4 _receptor in congenital heart block (CHB) associated to a systemic autoimmune response in the mother [10]. In neonatal lupus erythematosus (NLE), autoantibodies against ribonucleoproteins 48-kDa SSB/La, 52-kDa SSA/Ro (Ro52) and 60-kDa SSA/Ro (Ro60) have received more attention, since they have been shown to be strongly associated with autoimmune responses involved in symptoms like CHB [11]. It has been postulated that the transfer of maternal anti-Ro52 antibodies from mother to the fetus is responsible for the symptoms of NLE. In a previous study, we managed to induce NLE symptoms in a mouse model [12]. This has prompted us to further explore the expression of different 5-HT_4 _receptor isoforms in BALB/c mice during embryogenesis. Meanwhile, we searched for the period when anti-receptor antibodies could induce the observed abnormalities.

## Results and Discussion

### 1. Production of anti-peptide antibodies

**Figure 1:**
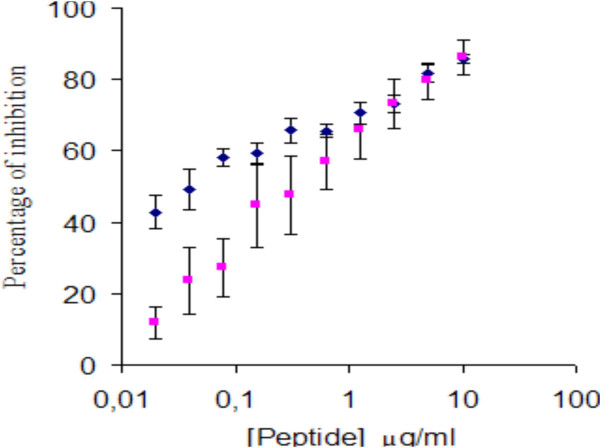
Inhibition enzyme immunoassay showing the specificity of anti-Y23F (◆) and anti-C7F (■) antibodies for their corresponding peptides. Mean values ± SD of the inhibition percentage obtained with increasing amount of peptides are represented.

**Figure 2:**
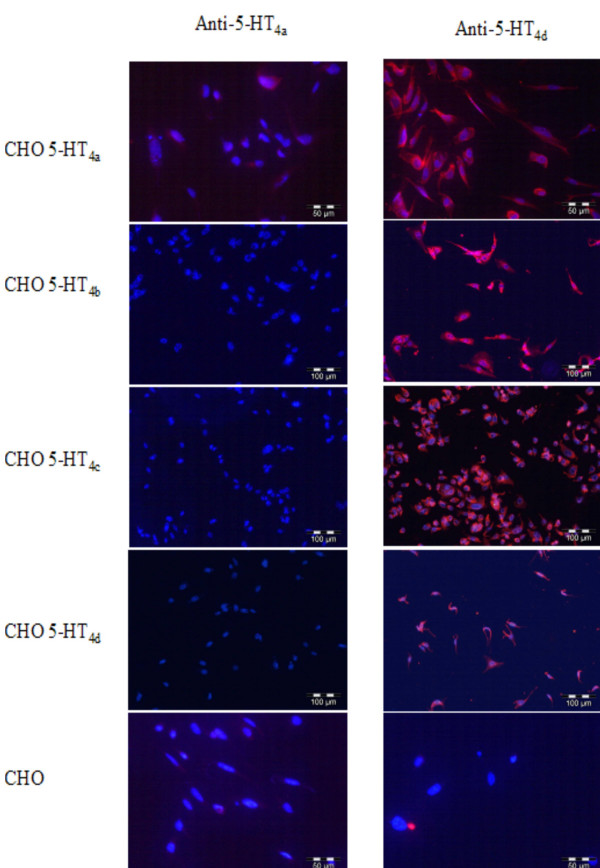
Specificity of anti-5-HT_4(a) _and anti-5-HT_4(d) _antibodies for their corresponding isoforms transiently expressed in CHO cells. Merge of DAPI nuclear staining and immunolabelling are shown. Anti-5-HT_4(a) _antibodies bind only their corresponding isoform, while anti-5-HT_4(d) _binds all isoforms (5-HT_4(a), (b), (c) and (d)_). No labelling was obtained with non -transfected cells.

Peptides derived from C-terminal ends of 5-HT_4 _receptor isoforms (Y23F and carrier- C7F) were highly immunogenic. The anti-5-HT_4(a) _and 5-HT_4(d) _antibodies were able to recognize both their homologue peptide in ELISA. The specificity of the recognition was tested by inhibition immunoassay (Figure 1). No cross-reactivity between the different anti-isoforms antibodies was detected in ELISA (data not shown). The antibodies recognized their corresponding receptor isoforms in immunocytofluorescence performed on transfected CHO cells (Figure 2). On transfected CHO cells, anti- 5-HT_4(a) _antibodies were specific for their corresponding isoform while anti-5-HT_4(d) _ones recognized 5-HT_4(a), (b), (c) and (d) _isoforms.

### 2. Expression of receptor isoforms

**Figure 3:**
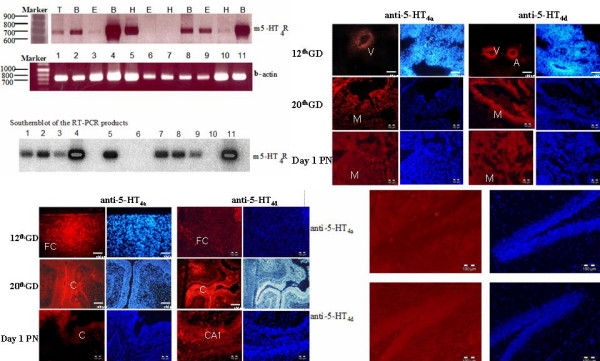
A) Expression analysis of 5-HT_4 _receptor transcripts in embryonic and post-natal mouse tissues. RT-PCR analysis was performed with RNA extracted from various mouse tissues (the brain (B), the eye (E), the heart (H), and the trunk (T). The 12^th^, 16^th^, 20^th ^GD and 1 DPN are represented in lane 1–2, 3–5, 6–8 and 9–11 respectively. The PCR products were analysed on a 1.5% agarose gel and photographs of the ethidium bromide stained gels are shown (upper panel). The PCR primers used for this analysis and expected length of the PCR products are described in Materials and Methods. A positive control was performed with β-actin primers on mRNA samples treated with reverse transcriptase. This figure is representative of three separate determinations of mouse 5-HT_4 _receptor mRNA expression obtained by RT-PCR. The specificity of the PCR products were analysed by Southern blotting using a ^32^P-5'-end-labeled internal primer (lower panel). A 3 h exposure of the autoradiogram is shown. Positions of the molecular weight markers are indicated in bp. m5-HT_4_R, mouse 5-HT_4 _receptor; Marker, molecular weight marker. 3B-D) Normal receptor expression at protein level detected using anti-5-HT_4(a) _and anti-5-HT_4(d) _antibodies in heart (B) atrium (a) myocardium (M) ventricle (V) brain, (C) in cortex (c) hippocampus (*CA*_1_) frontal cortex (*FC*), during different embryonic periods and at birth. Figure 3D shows the normal expression of 5-HT_4 _receptor in hippocampus of adult BALB/c mouse. Immunolabelling is shown in the left panel and DAPI nuclear coloration in the right one.

Embryos from 8 pregnant BALB/c mice gestation days (GD) 8^th^, 12^th^, 16^th^, 18^th^, 20^th ^and one day postnatal (DPN) were collected. These were used to explore the expression of 5-HT_4 _receptor isoforms both at the mRNA (RT-PCR) and protein levels. RT-PCR experiments revealed the increased expression of 5-HT_4 _receptor isoforms from day 12 of gestation to day 1 postnatal in brain. Receptor mRNA expression in the heart decreased gradually and disappeared at birth (Figure 3a). The latter was confirmed at the protein level where the expression of both isoforms matched the expressed 5-HT_4 _receptor mRNA (Figure 3b and 3c). In adult mouse brain we detected both anti-5-HT_4(a) _and anti-5-HT_4(d)_. Interestingly, the 5-HT_4(a) _receptor is absent in dentate gyrus of adult mouse hippocampus (Figure 3d).

### 3. *In vivo *effect of anti-5-HT_4 _antibodies

In order to highlight the role of 5-HT_4 _receptor on normal development of the embryos, we sought to immunomodulate the activity of the receptor in BALB/c mice embryos, through the passage of maternal anti-receptor antibodies induced with a peptide corresponding to the second extracellular loop of the 5-HT_4 _receptor. Pregnant mice at 8^th^, 12^th^, 18^th ^GD and one DPN were sacrificed and the embryos were counted and collected for morphological studies.

**Figure 4:**
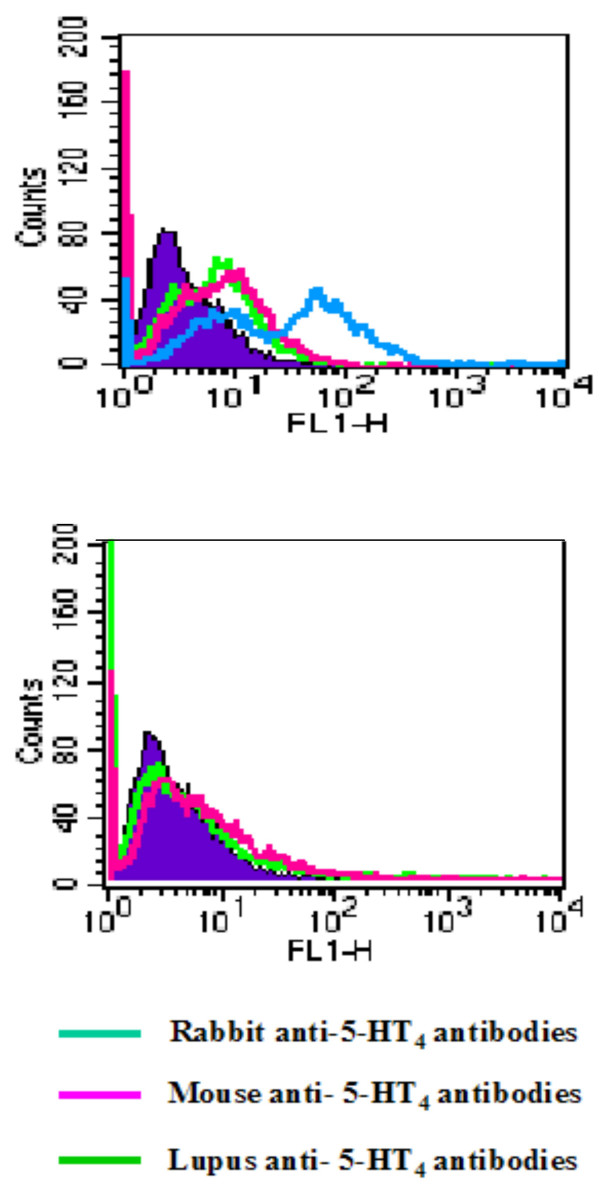
Showing the recognition of 5-HT_4(g) _receptor expressed in CHO cells (upper figure)using sera from immunized mouse with anti-5-HT_4 _antibodies (red), and positive controls, i.e. affinity-purified polyclonal rabbit anti-5-HT_4 _antibodies (blue) as well as affinity purified human anti-5-HT_4 _antibodies from a patient with lupus (green). The same antibodies showed no recognition of M_2 _muscarinic receptor expressed in CHO cells (lower figure).

**Figure 5:**
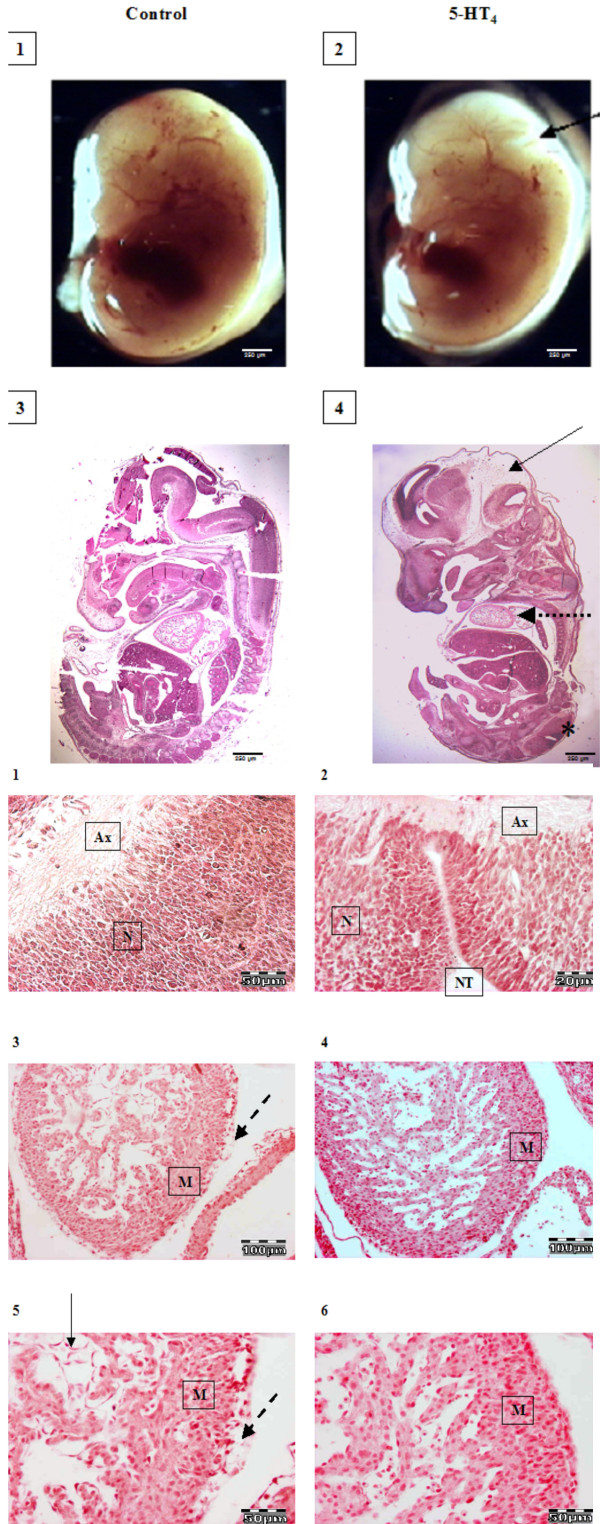
Morphological (A) and histological (B, C and D) studies on fetus from BALB/c mice immunized either with G21V (right) or with CFA (left). Note the difference in size (A2), delayed neuronal folding (A2 & 4), absence of hindbrain (arrow) and open neuronal tube (*) in fetus from mouse with anti-5-HT_4 _antibodies. Figures 5B 1–6 are the magnifications of figures 5A3 and 4 in order to highlight the abnormalities. Figure 5B1 shows the normal neuronal tube and 5B2 shows the open neuronal tube in control fetus and fetus from mouse with anti-5-HT_4 _antibodies (NT), axons (*Ax*) neurons (*N*). In Figure 5B3 and 5B4 foetal heart tissues, from control (5B3) and from mouse with anti-5-HT_4 _antibodies (5B4) are shown. Dotted arrow shows size and form abnormalities. Figure 5B5 and 6 show the higher magnification of the heart from control fetus (5B5) and fetus from mouse with anti-5-HT_4 _antibodies (5B6). The heart from the fetus in right panel is symmetric (5B4) compared to the fetus from the control mouse (5B3), where its heart is asymmetric with distinct and normal formation of apex. Myocardium (*M*), Pericardium (doted arrow), fibroblast (arrow)

All female mice immunized with G21V developed high-titre anti-peptide antibodies. Serum from the mouse having a fetus with spina bifida, recognized the 5-HT_4 _receptor by flow cytometry analyses performed on CHO cells transfected with 5-HT_4(g) _receptor (Figure 4). The same antibodies did not recognize any expression protein on CH0 cells transfected with M_2 _muscarinic receptor. Although immunized mice were all pregnant between 4 to 8 days post-mating, no pups were delivered at 21^st ^day post-fertility. Mice had to be mated more than three times over a period of 2 months for a successful pregnancy. This was probably due to the presence of anti-5-HT_4 _receptor antibodies. The fact that the female BALB/c mice had circulating anti-peptide antibodies did not alter their fertility but jeopardized the normal development of embryos. The latter was confirmed by the abnormal number of embryos collected at different periods of gestation. In addition we have also observed and documented different symptoms such as growth delay, spina bifida, arrhythmia and ataxia, which all started from the 12^th ^gestation day to 1 day PN (Table 1 and Figure 5a, b). Control mice (n = 5) gave birth to normal number of pups 21 days after the first mating (14 pups in total). The number of fetus in different embryonic periods was also normal i.e. 7 embryo in average (20 fetus in total). No abnormalities were observed neither in the embryos nor in the fetuses of the control did female mice, which were administer only CFA and mBSA.

**Table 1:** The outcome of mated mice immunised with G21V peptide

**Mouse**	**Titre**	**Fetus number**	**Observed alteration**	**Period**
1	1/200	7	1 growth delay	12 GD
2	1/1600	7	1 spina bifida	12 GD
3	1/1600	2	1 arrhythmia and 1 growth delay	18 GD
4	1/800	2	1 ataxia	1 DPN
5	1/100	6	1 arrhythmia and 1 growth delay	1 DPN

Summary of the outcome at different development intervals from female BALB/c mice immunized with G21V peptide. There is a relation between anti-G21V antibodies titre and number of fetuses/pups.

Table 2 summarises the titer of circulating anti-receptor antibodies, number of pups and abnormalities observed.

**Table 2:** Peptide sequences derived from human 5-HT_4 _receptors

Protein	Peptide	Sequence
5-HT_4 _(165–185)	G21V	GIIDLIEKRKFNQNSNSTYCV
5-HT_4(a) _(365–387)	Y23F	(Y)GHHQELEKLPIHNDPESLESCF
5-HT_4(d) _(354–360)	C7F	(C)THVLRF

Peptide residues covering second extracellular loop of serotoninergic 5-HT_4 _receptor (G21V), and residues covering C-terminal end of 5-HT_4(a) _(Y23F) and 5-HT_4(d) _(C7F).

**Figure 6:**
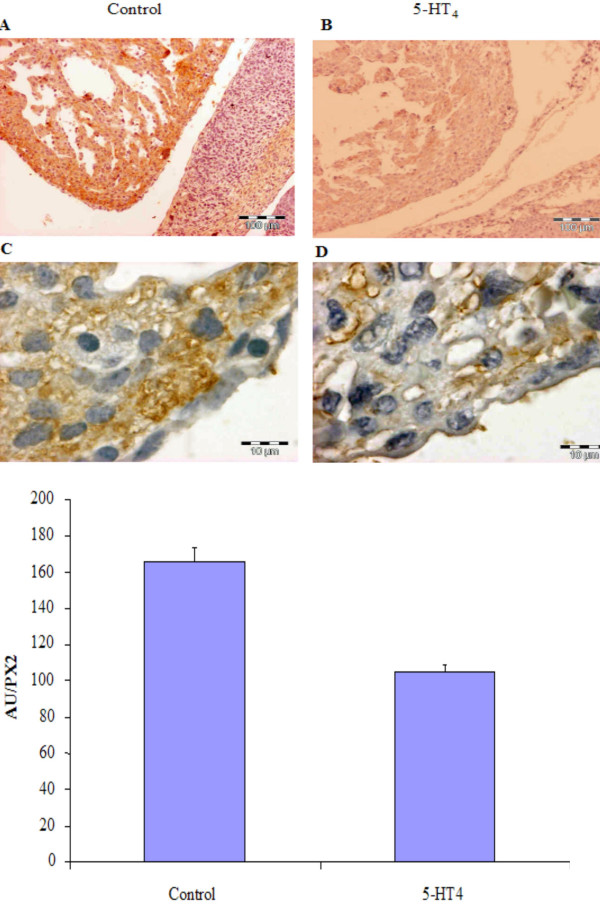
(A) Shows in heart decreased fibroblast immunoreactivity in fetus from a mouse with anti-5-HT_4 _antibodies. Hearts from a control fetus is shown in A (100×) and D (1000×) and from fetus affected by anti-5-HT_4 _antibody is shown in B (100×) and D (1000×). The presence of immunocomplex between anti-αSMA antibodies and heart tissue is revealed with DAB (orange colour in A and B) and counterstained with hematoxyline (Harris) as it shown in higher magnification in C and D. Figure 6B. Represents the estimated immunoreactivity as measured by densitometry in heart tissues from control fetus and fetus from mouse with anti-5-HT_4 _antibodies (expressed in absorbance unit/pixel area, AU/px^2^)

Morphological studies showed either growth delay or late development of foetal hindbrain at 12^th ^GD (Figures 5A, 5B). Histological studies showed, besides the presence of spina bifida, delayed foetal neuronal differentiation in embryos from the mother with circulating anti-5-HT_4 _antibodies. In the heart of the same embryo symmetric form of the heart was observed with pronounced hyperplasia and decreased fibroblast density (Figure 5B). The latter was confirmed with decreased immunoreactivity for α-SMA in the heart of the embryos from the mouse with circulating anti-5-HT_4 _antibodies (Figure 6A, 6B)

## Conclusion

Up to now there is no information on the distribution of the 5-HT_4 _receptor isoforms at the protein level. The fact that there are at least 10 different 5-HT_4 _receptor isoforms and mRNA expression in both central and peripheral organs, point to the importance of this family of receptors in the maintenance of normal cellular activity. Although 5-HT_4 _receptor has been reported to be involved in memory and learning as well as gastrointestinal function, less or almost nothing is known about its role in embryogenesis.

The importance of the embryonic serotoninergic system in central nervous and cardiovascular functions has been largely described [15-20]. In early mouse embryogenesis, maternal serotonin (5-HT) activates different 5-HT receptors to control gene expression, migration and proliferation of neuronal crest and neuronal-crest derived cells [21-25]. Although there is enough evidence fortifying the 5-HT hypothesis in embryogenesis, no major abnormalities were found in 5-HT_4 _receptor knockout mice. One has just observed an attenuated response to stress and novelty as well as hypersensitivity to seizures [31]. There are many knockout and transgenic mouse models with such phenotypes. For instance null mutation of α4 subunit nicotinic cholinergic receptor increases the susceptibility to proconvulsant-induced seizures [32]. This is also the case for 5-HT_1A _receptor, prion protein, superoxide dismutase, tumor necrosis factor-alpha [32-34] and many others. Blocking the expression

of 5-HT receptors using traditional knockout method gives rise to compensatory mechanism that occur during the constitutive deletion of the receptor throughout the lifespan of the animal. Furthermore, the inherent complexity in the pattern of expression and in the function of some genes and gene products contributes more to compensation and hidden developmental roles [35]. On the contrary by using alternative methods like blocking the expression of the receptor in restricted periods of time one circumvents the compensations [32]. The latter is also possible by modulating the protein activity at the cell membrane rather than blocking the function of the gene. Given the pathophysiological complexity of these conditions and involvement of many other factors one cannot explain these abnormal conditions with the lack of 5-HT_4 _receptors. One plausible explanation to the latter is the expanded variety of the 5-HT receptor family. Loosing one receptor subtype or isoform probably leads to a compensation mechanism conducted by other members of the family here probably 5-HT_6 _serotoninergic receptor. The only 5-HT receptor knockout with pathophysiological consequences is the 5-HT_2(b) _receptor. 5-HT_2(b) _receptor knockout mice embryos developed cardiac hypoplasia [24]. The receptor's importance in regulating cardiac activity was reported two years later [26]. Our group has shown in pups of BALB/c mice that maternal anti-5-HT_4 _antibodies caused fetal cardiac hyperplasia and arrhythmia [12]. In the central nervous system, 5-HT_4 _receptor isoform mRNAs were found in almost all brain structures. However, up to now neither the receptor expression nor its function during the fetal period was studied.

In the first part of this study, we demonstrated the specificity of the anti-peptide antibodies for their corresponding peptide. Anti-5HT_4(a) _was specific for its isoform as it did not cross react with any of the other receptor isoforms tested, i.e. 5-HT_4(b), (c) and (d)_. On the contrary, 5-HT_4(d) _antibodies, besides binding its corresponding peptide could also recognize all the other isoforms including 5-HT_4(a)_. This was not due to a cross reactivity but to their specificity, since the C- terminal end of 5-HT_4(d) _is common between 5-HT_4(a), (b), (c) and (d)_. We report here the presence of cardiac isoforms 5-HT_4(a) _in embryos from BALB/c mice at GD 8^th ^(data not shown), 12^th^, 18^th ^and one day postnatal. Since the anti-5-HT_4(d) _antibodies are able to recognize other already mentioned isoforms, we can only be sure about the expression of 5-HT_4(a) _in the heart and brain. Whether there are other isoforms expressed during embryogenesis remains elusive. However 5-HT_4(a) _receptor was found both in fetal brain and heart. Both 5-HT_4(a) _and 5-HT_4(d) _receptor isoforms were expressed in brain, at 1 DP, but we could not detect any signal in the pups' heart for the two explored isoforms. One remains, however, puzzled of the receptor's disappearance at 1 DP. This could be explained by the progressive appearance of adrenergic receptors. Indeed, both α- and β-adrenergic receptors are already detected at 12^th ^GD, and, contrary to 5-HT_4 _receptor, activity of β-adrenergic receptor in mouse heart increases significantly until birth [27].

In the second part of our study, we observed that all female BALB/c mice immunized with peptide derived from the second extracellular loop of 5-HT_4 _receptor developed anti-peptide antibodies that could recognize membranes from 5-HT_4(g) _transfected CHO cells. Although the mice were all fertile, they had to be mated several times before developing a successful pregnancy. All mice in the control group gave birth 21 days post mating. In immunized mice, the number of pups for the first pregnancy was between 5 and 7, which was similar to the control group. However, the number of fetuses of immunized mice decreased from 12^th ^to 18^th ^GD (7 to 2, respectively). This was in accordance with the titer of circulating antibodies in the mothers. The latter was also observed in two littermates, where one mouse gave birth to 2 and the other to 6 pups. Here we observed again that the titer of circulating antibodies for the mother with just two pups was 7 times higher than the mouse with 6 pups.

In this study, we demonstrated that 5-HT_4 _receptors are expressed already at the 8^th ^GD (in ectoderm), at least for the 5-HT_4(a) _isoform. We also provided evidence for the functionality of the anti-receptor antibodies, since 1) they decreased fertility as shown by the increased number of mating for a successful litter, 2) they decreased the number of fetuses and pups in relation with the titer of circulating antibodies, 3) they induced morphological and functional abnormalities both in peripheral and central organs. In our earlier study [12] we demonstrated that anti-5-HT_4 _antibodies passively transferred from mother to the pups could induce atrioventricular block type I and II (AVB), tremor and skin lesions. Here we confirmed the presence of malformation in some cases and could correlate its incidence with the expression of the receptor and titre of the anti-receptor antibodies. This means that at high concentration of transferred anti-receptor antibodies the chance of malformation is considerably higher, since 21-days post mating either the number of pups decreased or no labour was issued. The effect of anti-5-HT_4 _antibodies could be through their binding to at least 5-HT_4(a) _receptor isoform, since it is already expressed from 8^th ^GD. It has been shown that antibodies against the second extracellular loop of the receptor block the effect of serotonin in human cardiomyocytes [10,28]. In embryonic heart muscle expression of vascular α-actin has been demonstrated. Alpha-actin acts as a precursor in immature contractile structures, when the embryonic heart just starts to beat, and might only gradually be replaced by the mature cardiac isoform [35-37]. For this reason we used α-smooth muscle actin as a marker for heart development. Our observation that myocardium of mouse fetus with circulating maternal anti-5-HT_4 _antibodies has less or no immunoreactivity for α-actin smooth muscle could be due to delayed maturation of fetal myocardium in immunized animals. However although the presences of anti-Ro52 autoantibodies cross reacting with SEL of 5-HT_4 _receptors shows a good correlation with the prevalence for neonatal lupus their pathogenic role cannot be generalized. We have shown this in our previous work [38]. This could be due to at one hand that the tested sera were from pretreated pregnant women with lupus. In no studies yet published the blood samples are collected following the same period of pregnancy. Beside the important fluctuation of circulating autoantibodies makes the exploration even harder. On the other hand we don't have any idea about the expression of the 5-HT_4 _receptor during human embryogenesis. Therefore we cannot draw any general conclusion. In mice (BALB/c) with no known autoimmune genetic background, therefore requiring no treatment, only the presence of 5-HT_4 _receptor antibodies induced anomalies in fetus, which coincide with the receptor expression. Here we could postulate that the SEL of 5-HT_4 _receptor could be a pathogenic target for autoantibodies

Although the effect of the antibodies seems to be through the inhibition of the serotonin signal we however have no evidence for its cellular mechanism.

Finally, we also show that immunomodulation of 5-HT_4 _receptor activity, during embryogenesis, is an honorable alternative to the gene knockout technique. Since the receptor is already expressed and functional, modulating or inhibiting its activity will not activate any genetically compensatory mechanism. Therefore, its activity and physiological role can be explored independently.

Taken together, our results demonstrate that 5-HT_4 _receptors are crucial for embryonic development of the cardiovascular and central nervous systems in BALB/c mice. Coincidence of anti-receptor antibodies and expression of 5-HT_4 _receptor results in anatomical abnormalities. Further studies are needed to highlight the function of different receptor isoforms during embryogenesis.

## Methods

### 1. Animals

Female adult rabbits (n = 4), female (n = 15) and male (n = 16) BALB/c mice were purchased from Janvier (Tours, France). The animals were kept on a 12:12-h light-dark cycle (lights on at 6:00 AM) at 21 ± 1°C ambient temperature. Water and food were available ad libitum throughout the experiments.

### 2. Peptides

A peptide corresponding to the second extracellular loop of h5-HT_4 _receptor (G21V) [10,9], two other peptides corresponding to C- terminal ends of the 5-HT_4 _isoforms h5-HT_4(a) _(Y23F) and h5-HT_4(d) _(C7F) were synthesized as previously described (Table 2) [10,29]. Their structure and purity were checked by mass-spectrometry and HPLC.

### 3. Induction of anti-peptide antibodies

Eight female adult rabbits (2.5–2.7 kg) were immunized with either Y23F or C7F peptides. Four female rabbits were immunized with 200 μg of peptide Y23F with 1 mg of mBSA. Prime injections were given in CFA. The peptide C7F was conjugated to BSA using SBA prior to administration to other four rabbits. The peptide was coupled according to the following procedure. All sulphydryl groups on BSA (4 mg) were blocked with 0.5 M iodoacetamide in a coupling buffer (pH = 7) composed of K_2_HPO_4 _(100 mM), EDTA (1 mM) and sodium azide (0.02%). The mixture was dialyzed 3 times 1 liter, against the coupling buffer without sodium azide. Thereafter 30 mg (0.13 mmol) of SBA was added to 4 mg of saturated BSA in the presence of 0.25 ml of dimethylformamide and diluted with coupling buffer to the final volume of 3 ml. SBA conjugated BSA was separated from the unconjugated ones on a G-25 Sephadex column (2.2 × 27 cm) and dialyzed against coupling buffer (3 times 1 liter). Finally 3.4 mg/ml of activated BSA molecule was mixed with 1.6 mg of C7F peptide. It was then dialyzed against the coupling buffer and stored at -20°C. Rabbits were immunized with an equivalent of 200 μg of BSA conjugated C7F in presence of CFA. Thereafter rabbits received 3 booster injections with three weeks interval where CFA was replaced with Incomplete Freund Adjuvant (IFA). They were bled and the presence of anti-peptide antibodies was checked.

Female BALB/c mice (n = 5) were immunized with 15 μg/mouse of peptide G21V together with 100 μg/mouse mBSA supplemented with PBS/CFA (50% v/v). Two booster injections were thereafter administered with 3 weeks intervals, where CFA was replaced with IFA. Control mice (n = 5) received just CFA and IFA. Mice were bled one week after each booster injection and sera were tested for the presence of the anti-peptide antibodies.

### 4. Affinity purification

Y23F was fixed on carboxyl activated ECH Sepharose beads (Amersham Pharmacia, Sweden), and C7F or G21V peptides were fixed on thiol activated Sepharose beads (Amersham Pharmacia, Sweden) according to the standard procedures. Immunoglobulins from the immunized rabbit sera as well as lupus patient plasmaphereses with positive activity on G21V peptide in ELISA, were precipitated with 33% saturated ammonium sulfate and extensively dialyzed over night against PBS. Immunoglobulins were thereafter diluted 10–15 times with filtered PBS, before passing through the affinity columns at 4°C for 3 h. After wash out, the anti-peptide polyclonal antibodies were eluted with 3 M KSCN and dialyzed immediately against 6 liters PBS at 4°C overnight.

### 5. Enzyme- linked immunosorbent assay (ELISA)

Microtitre plates (Falcon, Canada) were coated with 5 μg/ml of peptide (50μl/well) in coating buffer (carbonate buffer 0.1 M, pH = 9.6) for 1 h at 37°C. After saturation with 1% BSA in PBS supplemented with 0.1% Tween 20 (blocking buffer), the plates were incubated with increasing dilutions (10^-2 ^to 10^-6^) of sera from immunized and control rabbits for 1 h at 37°C. After several washes with PBS-Tween, the plates were allowed to react with affinity-purified goat anti-rabbit IgG, Fc fragment specific antibodies conjugated to horse-radish peroxidase for 1 h and revealed with 3,3',5,5'-tetramethyl benzidine in the presence of H_2_O_2_. Reaction was stopped with 1 N HCl and absorbance was read at 450 nm in a microplate reader. Inhibition immunoassay was performed as previously described [9]. Before using affinity purified rabbit polyclonal anti-5-HT_4a and d _antibodies in ELISA as described above, the antibodies were diluted 1/12800 in blocking buffer and incubated with decreasing concentrations (10 - 0.04 μg/ml) of either Y23F or C7F respectively for 1 h at 37°C.

### 6. Cell culture and transfection

Non-transfected CHO cells were cultivated in Dulbecco's Modified Eagle's Medium (DMEM) (Sigma Aldrich) supplemented with 10% bovine fetal serum (BFS), 100 U/ml penicillin, 100 μg/ml streptomycin. The cells were incubated in a humidified incubator at 37°C under an atmosphere of 5% CO_2_. Semi-confluent CHO cells were transfected as previously described by [30]. Briefly, transfection was performed using a mixture of the vector polyethyleneimine (PEI) and plasmids corresponding to h5-HT_4 _receptor isoforms (a) or (d) at a ratio of 20 nM PEI/mg of DNA. The cells were incubated for 5 h at 37°C with serum free DMEM supplemented with 100 U/ml penicillin, 100 μg/ml streptomycin. This was followed by incubation in BFS supplemented medium for 24 h.

### 7. Immunocytochemistry

CHO cells expressing the 5-HT_4 _receptor isoforms (a) and (d) as well as non-transfected cells were deposited on Superfrost Plus slides and fixed for 2 min in paraformaldehyde 4%. They were washed with PBS-Tween 20 (0.1%) and saturated with PBS supplemented with 0.1% Tween 20 and 0.1% BSA for 1 h at room temperature. They were incubated with 1/200 affinity purified anti-peptide antibodies corresponding to each isoform for 2 h at room temperature followed by 1 μg/ml 4',6-diamidino-2-phenylindole (DAPI) (Sigma Aldrich) and Alexa Fluor 594 goat anti-rabbit IgG (H+L) (Molecular Probes, Oregon, USA) diluted 1/500 for 1 h at the same temperature. The slides were mounted using DAKO Fluorescent mounting medium (DAKO CORPORATION, Carpinteria).

### 8. Flow cytometry analyses

Stably transfected 5-HT_4(g) _and M_2 _muscarinic CHO cells were cultured and growth to confluence separately in DMEM medium supplemented with 10% heat inactivated BFS. Twenty-four hours before experiment, cells were harvested with chilled Trypsin/EDTA washed and dispatched at a concentration of 10^5 ^cells/tube in pretreated FACS tubes, in order to prevent cell attachment on the tube wall. Affinity purified rabbit polyclonal anti-G21V (1/500), mouse sera (1/100) and affinity purified anti-G21V (1/50) from a lupus patient were incubated with the cells for 1 h in normal culture condition. Cells were washed 3 times with DMEM. They were then incubated with either FITC-conjugated goat anti-rabbit or FITC-conjugated goat anti-mouse or biotinylated goat anti-human antibodies in DMEM supplemented with 1% goat sera for 1 h in normal culture conditions. After 3 washes only tubes treated with anti-human antibodies were further incubated with FITC- conjugated streptavidin for 30 min in normal culture conditions.

Cells were analyzed by flow cytometry with a FACSCalibur^® ^. At least 10,000 events were acquired for each experiment using the CellQuest 3.3 software (Becton Dickinson, Pont de Claix, France). The data were processed with the same program using analyses option.

### 9. Reverse Transcription – Polymerase Chain Reaction (RT-PCR) and southern blot

Balb/c mice (n = 8) were mated and the fetuses were collected at 8^th^, 12^th^, 16^th ^18^th^, 20^th ^GD and 1 DPN. From the same litter, half of fetus was used for RT-PCR and the other half was frozen down for immunohistochemistry (see below). Total RNA was prepared from mouse tissues using the Trizol RNA purification system (Life Technologies Inc.). After purification, RNA was treated with DNase I (Life Technologies Inc.) and 5 μg of total RNA was then hybridized with oligo(dT) primer and reverse transcribed using Superscript reverse transcriptase II (Life Technologies Inc.). The resulting single strand cDNAs were used as templates in PCR reactions using a specific primer pair for the m5-HT_4_R (5'-CCTGTGCTGTATTTCCCTGG-3'; 5'-GAATGGGGGGTCTTTTGTAG-3'). This primer couple selective of the common part of mouse 5-HT_4 _receptor isoform allows the amplification of a single DNA band migrating at 688 bp (base pair). The sequence of β-actin primers has been previously described [5]. PCR reactions were performed using the following cycle conditions: denaturation for 1 min at 94°C, annealing for 30 s at 55°C, and extension for 1 min 30 sec at 72°C with a final extension step for 8 min at 72°C. The PCR products were electrophoresed on 1.5% agarose gel containing 0.01% ethidium bromide and photographed under U.V. irradiation. For Southern blot, PCR products were transferred to Zetaprobe membrane (Biorad). The following primer 5'-AGGATGATGAGGAAGGCACG-3' was labeled with ^32^P using the random primer DNA labeling kit (Life Technologies Inc.), and used as an internal probe for hybridization. Hybridization was performed at 57°C for 12 h in a Church buffer (0.5 M NaHPO_4 _pH 7.2, 1 mM EDTA, 7% SDS) containing 100 μg/ml of denatured salmon sperm DNA. The membrane was revealed using a phosphoimager (Storm, Molecular Dynamics).

### 10. Immunofluorescence and histochemistry

Frozen fetuses 12^th ^18^th ^GD, pups and adult brains were subjected to serial cuts at 5-μm thickness, using a Leica cryostat. After fixing the tissues with 1% paraformaldehyde for 5 min at room temperature, they were permeabilized in PBS at pH 7.4, supplemented with 0.1% Triton-X for 3 min. The tissue was saturated with blocking buffer (PBS, 1% BSA, 0.1%Tween) for 1 h at room temperature. Affinity purified anti-peptide antibodies corresponding to each isoform were incubated for 2 h (1/200). After three washes in PBS, the tissues were incubated for 1 h at room temperature with Alexa Fluor 594 goat anti-rabbit IgG (H+L) (1/500) and visualized under a Leica epifluorescence microscope and photographed.

Fetuses at 12^th ^GD were collected and fixed overnight in 4% formaldehyde/PBS solution. They were then dehydrated according to the standard procedures. Paraffin embedded fetuses were cut on the sagital axis at 4 μm mounted on superfrost plus slides. They were then dried over night at 57°C. Sections were rehydrated and saturated for 1 h in TBS-Tween 0.1% supplemented with 1% goat sera. Slides were thereafter incubated with anti- Actin, α smooth muscle (α-ASM) mouse monoclonal (Sigma-Aldrich St Louis). After three washes Vectastain ABC KIT goat anti-mouse immunoglobulins (Vector laboratories, Burlingame, CA) was used according to the manufacturer recommendations. Finally after extensive washes (5 times in TBS Tween) the bound antibodies were revealed using DAB chromogen KIT (DakoCystomation, Carpinteria, CA). The slides were counter stained with hematoxylin for 20 sec and washed for 15 min under demineralized water. They were then dehydrated and mounted according to the standard procedures. The immunoreactivity in fetal heart was quantified in gray scale using MacBas densitometry program on 5 serial slices and 10 different regions for each slide.

### 11. Histology

Fetuses at 12^th ^GD were collected and fixed over night in 4% formaldehyde/PBS solution. They were then dehydrated according to the standard procedures. Paraffin embedded fetuses were cut on the sagital axis at 4 μm and subjected to standard hematoxilin/eosin coloration.

## List of Abbreviations

Atrio-ventricular block (AVB), Chinese Hamster Ovary (CHO), Complete Freund's Adjuvant (CFA), congenital heart block (CHB), 4',6-diamidino-2-phenylindole (DAPI), Dulbecco's Modified Eagle's Medium (DMEM), day post natal (DPN) Enzyme- Linked immunosorbent assay (ELISA), ethylenendiaminetetraacetic acid (EDTA), gestation days (GD), human 5-HT_4 _receptor (h5-HT_4_), Incomplete Freund Adjuvant (IFA), mouse 5-HT_4 _receptor (m5-HT_4_), methylated bovine serum albumin (mBSA), neonatal lupus erythematosus (NLE), one day postnatal (DPN), polyethyleneimine (PEI), Reverse Transcription – Polymerase Chain Reaction (RT-PCR), N-succinimidyl bromoacetate (SBA), second extracellular loop (SEL).

## Authors' contributions

All authors read and approved the final manuscript.

RK has performed ELISA, Western blot and immunohistological experiments.

SG has also performed ELISA, contributed to the in vivo studies, and explored the fetal abnormalities.

FL has performed mRNA studies.

RF has revised critically and contributed with important intellectual input regarding this manuscript.

SM has revised critically and contributed with important intellectual input regarding this manuscript.

JH has made substantial contributions to conception and design of the manuscript

PE has induced antibodies, performed histological studies, conducted the experimental part and made substantial contributions to conception and design of the manuscript.
